# Healthcare workers’ SARS-CoV-2 infection rates during the second wave of the pandemic: follow-up study

**DOI:** 10.5271/sjweh.4049

**Published:** 2022-10-01

**Authors:** Anne Mette Würtz, Martin B Kinnerup, Kirsten Pugdahl, Vivi Schlünssen, Jesper Medom Vestergaard, Kent Nielsen, Christine Cramer, Jens Peter Bonde, Karin Biering, Ole Carstensen, Karoline Kærgaard Hansen, Annett Dalbøge, Esben Meulengracht Flachs, Mette Lausten Hansen, Ane Marie Thulstrup, Else Toft Würtz, Mona Kjærsgaard, Mette Wulf Christensen, Henrik Albert Kolstad

**Affiliations:** 1 Department of Public Health, Work, Environment and Health, Danish Ramazzini Centre, Aarhus University, Aarhus C, Denmark; 2 Department of Occupational Medicine, Danish Ramazzini Centre, Aarhus University Hospital, Denmark; 3 Department of Occupational Medicine, Danish Ramazzini Centre, Goedstrup Hospital, Herning, Denmark; 4 Department of Occupational and Environmental Medicine, Bispebjerg and Frederiksberg Hospital, University of Copenhagen, Copenhagen, Denmark; 5 Department of Clinical Microbiology, Aarhus University Hospital, Aarhus, Denmark; 6 Institute of Clinical Medicine, Aarhus University, Aarhus, Denmark

**Keywords:** coronavirus, COVID-19, epidemiology, infectious disease, longitudinal study, loss of taste and smell, occupational safety, PCR, polymerase chain reaction, risk factor

## Abstract

**Objectives:**

This study aimed to assess if, during the second wave of the COVID-19 pandemic, healthcare workers had increased severe acute respiratory syndrome coronavirus 2 (SARS-CoV-2) infection rates, following close contact with patients, co-workers and persons outside work with COVID-19.

**Methods:**

A follow-up study of 5985 healthcare workers from Denmark was conducted between November 2020 and April 2021 and provided day-to-day information on COVID-19 contacts. SARS-CoV-2 infection was defined by the first positive polymerase chain reaction (PCR) test ever. Data was analyzed in multivariable Poisson regression models.

**Results:**

The SARS-CoV-2 infection rates following close contact 3–7 days earlier with patients, co-workers and persons outside work with COVID-19 were 153.7, 240.8, and 728.1 per 100 000 person-days, respectively. This corresponded with age, sex, month, number of PCR tests and mutually adjusted incidence rate ratios of 3.17 [40 cases, 95% confidence interval (CI) 2.15–4.66], 2.54 (10 cases, 95% CI 1.30–4.96) and 17.79 (35 cases, 95% CI 12.05–26.28). The risk of SARS-CoV-2 infection was thus lower, but the absolute numbers affected was higher following COVID-19 contact at work than COVID-19 contact off work.

**Conclusions:**

Despite strong focus on preventive measures during the second wave of the pandemic, healthcare workers were still at increased risk of SARS-CoV-2 infection when in close contact with patients or co-workers with COVID-19. There is a need for increased focus on infection control measures in order to secure healthcare workers’ health and reduce transmission into the community during ongoing and future waves of SARS-CoV-2 and other infections.

The first wave of the SARS-CoV-2 pandemic was globally characterized by widespread lack of personal protective equipment (PPE), confusing PPE guidelines and lack of SARS-CoV-2 testing and contact tracing (1). Healthcare workers were at highly increased risk and mortality of COVID-19 (2–6). From January to October 2020, healthcare workers from four UK teaching hospitals exposed to patients and healthcare workers infected with SARS-CoV-2 had infection rates of 0.8 and 0.6 per 1000 person-days at risk, respectively, well above the background rate of 0.1 per 1000 person-days at risk (7). From March to April 2020, front-line healthcare workers in the UK and USA reporting adequate PPE use when in direct contact with COVID-19 patients showed a five-fold increased self-reported positive polymerase chain reaction (PCR) testing rate for SARS-CoV-2 of 553 per 100 000 (2). Increased SARS-CoV-2 seropositivity was reported among healthcare workers in close contact with patients (8–11), co-workers (8, 9), household members and other persons outside work with COVID-19 (8, 9, 12–15), but not consistently (13–16).

A considerable increase in preventive measures was initiated in multiple countries including Denmark (13), and it was expected that the pandemic afflicting so many healthcare workers was brought under control during the second wave. Our main objective was to study if, during the second wave of the COVID-19 pandemic, healthcare workers had increased SARS-CoV-2 infection rates following close contact with patients, co-workers and persons outside work with COVID-19.

## Methods

### Study design and population

This is a dynamic follow-up study with day-to-day self-reported information on COVID-19 contacts. Outcome is incident SARS-CoV-2 infection. Person-day at risk is the unit of analysis. The study population comprised healthcare workers at hospitals and related technical, administrative and other staff of the Central Denmark Region (hereafter referred to as healthcare workers).

### General surveillance and infection control recommendations

PCR testing for SARS-CoV-2 was freely accessible at no cost for all Danish citizens independent of symptoms. All hospital workers with any patient contact were urged to be PCR tested bi-weekly until 26 January 2021, thereafter weekly. PCR test results were provided on average 24–36 hours after sample collection. SARS-CoV-2 infection rates in the second wave of the pandemic peaked in Denmark on 16 December 2020, with 4387 PCR-verified cases in a population of 5 771 877 citizens.

All healthcare workers were instructed to follow general guidelines for infection control and wear surgical masks in all indoor areas with public or patient access and maintain physical distance to other persons whenever possible. All workers with non-critical functions were sent home on 11 December 2021, and for the remaining study period.

During care for patients diagnosed with or under suspicion of COVID-19, all staff were instructed to wear a fluid-repellent disposable gown with long sleeves, disposable medical gloves, surgical mask and protective glasses or visor. Moreover, during procedures with risk of aerosol generation (eg, high flow oxygen therapy) the surgical mask should be replaced by a filtering face piece 2 or 3 (FFP2, FFP3) respirator. The healthcare workers were instructed how to do a positive pressure seal check of the respirators, but no formal fit test was done as recommended by Center for Disease Control (18). There was sufficient supply of PPE during the study period.

Following close contact with persons diagnosed with COVID-19 without prescribed PPE for ≥15 minutes, individuals were required to go into self-isolation and be PCR tested at day four and six. Self-isolation could be cancelled following two negative tests or, in case of a positive test, 48 hours after symptom cessation or seven days after the positive test if asymptomatic. Detailed infection control for COVID-19 for employees of the Central Denmark Region during the COVID-19 pandemic can be found in the supplementary material (www.sjweh.fi/article/4049).

### Exposure assessment of COVID-19 contacts

Each day during follow-up at 15:30 hours, study participants received a text message linking to a questionnaire. They were asked to report any incident of close contact within a one-meter distance with patients and persons outside work with COVID-19 during the current and the previous 1–2 and 3–4 days. Participants were also asked to report incidents of close contact with co-workers with COVID-19 during the previous 1–2 and 3–4 days, but not the current day, because co-workers with known COVID-19 would not be present at work.

We classified each day of follow-up as exposed to contact with COVID-19 patients if participants reported such contact at least once during the previous 3–7 days. Otherwise, each day of follow up was classified with no close contact with COVID-19 patients if participants reported this for ≥3 days during the previous 3–7 days. Days of follow-up not fulfilling these two criteria were classified with unknown close contact with COVID-19 patients. A similar approach was used to assign exposure status following close contact with co-workers and persons outside work. Thus, each day of follow-up was classified as exposed (yes, no, unknown) to patients, co-workers and persons outside work with COVID-19. To account for the incubation period, we decided to focus on the 5-day exposure window 3–7 days earlier (19).

### SARS-CoV-2 infection, vaccination and COVID-19 symptoms

The primary outcome measure was incident SARS-CoV-2 infection defined as the first positive PCR test ever recorded in a regional register with complete coverage of all tests conducted in the population since 27 February 2020. A regional register also provided information about all COVID-19 vaccinations since 27 December 2020. As a secondary outcome measure, we included first report of loss of taste and smell as asked for in the daily questionnaire because this was a key symptom of COVID-19 during the early waves of the pandemic and should be unaffected by potential biases related to being PCR tested for SARS-CoV-2 (20).

### Population characteristics

Information on age, sex, occupation and department of employment was obtained from the personnel records of the Central Denmark Region. At baseline, participants reported information on smoking, height and weight that allowed calculation of body mass index (BMI), airways disease (chronic obstructive pulmonary disease, asthma, rhinitis). Participants reported non-compliance with PPE guidelines in the daily questionnaire by responding to the following two questions: “Has there within the last 24 hours been situations where you did not use the recommended personal protective equipment? If yes, during which tasks did you not use the recommended personal protective equipment?”.

### Statistical analyses

Study participants were followed daily from seven days after the first daily questionnaire response – 25 November 2020 – at the earliest, until first positive test for SARS-CoV-2, seven days after full vaccination (21) or 30 April 2021. Each day of follow-up was classified according to close contact (yes, no, unknown) with patients, co-workers and persons outside work with COVID-19 according to the previously defined criteria. Participants may have experienced all contact forms several times during follow-up and thus move in and out of exposures.

Study population characteristics were described for the person-days at risk. Sex, age, occupation, department, smoking, BMI, and lung disease were reported at baseline and did not vary during follow up. PCR testing, on the other hand like close contact with patients, co-workers and persons outside work with COVID-19, varied day-to-day, and we therefore reported PCR testing for three time windows: 1–2, 3–7 and ≥8 days earlier reflecting time after, during, and before the 5-day COVID-19 exposure window.

We used generalized linear models with log-link assuming a Poisson distribution with person-days as offset representing the time at risk to derive incidence rate ratios (IRR) with 95% confidence intervals (CI) for SARS-CoV-2 infection following close contact versus no close contact with patients, co-workers and persons outside work with COVID-19. Adjusted IRR were mutually adjusted for the other types of COVID-19 contact, sex, age (continuous) and month (6 categories, November 2020–April 2021) as decided a priori. We furthermore adjusted for number of PCR tests made before, during, and after the 5-day exposure window (≥8, 3–7 and 1–2 days previously). However, this only affected IRR estimates marginally, and in the final models, we included the cumulative number of earlier PCR tests as a continuous variable. In the analyses, number of previous PCR tests and contact with patients, co-workers and persons outside work were treated as time-varying day-to-day.

We excluded person-days with missing information on close contact with patients, co-workers and persons outside work diagnosed with COVID-19. We abstained from imputing the missing values. This was because a high fraction of participants worked part time or irregular shifts with at least two days off work with no close contact with patients or co-workers at unpredictable days during a given week. Information on the covariates of the adjusted models were complete.

In a sub-analysis, we restricted the data to person-days at risk with close contact either with patients, co-workers or persons outside work with COVID-19, excluding person-days with combined close contacts. Based on this data, we estimated the IRR of SARS-CoV-2 infection following close contact with either patients or co-workers using close contact with persons outside work as the reference.

Analyses of loss of taste and smell followed a similar setup as analyses of SARS-CoV-2 infection, but we did not censor subjects when testing PCR positive for SARS-CoV-2 and did not include number of earlier PCR tests in the adjusted models.

In sensitivity analyses of possible differential recall of close COVID-19 contacts, we excluded contact information obtained after a given day of follow-up (ie, based on questionnaire reports for the previous 1–2 and 3–4 days), when PCR test results were available for the participants. This excluded all information on close contact with co-workers with COVID-19 because this was only reported for the previous 1–2 and 3–4 days.

## Results

A total of 26 089 healthcare workers were invited to the study on 17 November 2020. After excluding 724 who tested positive for SARS-CoV-2 before the start of follow-up, 25 365 healthcare workers (3 253 671 person-days) were candidates for inclusion and 6337 (753 607 person-days) participated (table 1). After excluding person-days with missing information on close contact with patients, co-workers or persons outside work with COVID-19, the study population included 5985 healthcare workers providing 514 165 person-days at risk. The daily testing rates were 5.5% for the invited population and 7.1% for the study population. Altogether, 448 748 daily questionnaire responses were collected from the study population during follow-up, corresponding with an 87.3% coverage. SARS-CoV-2 infection rates in the invited population and the study population were 28.6 and 30.9 per 100 000 person-days.

**Table 1:** Study profile.

Populations	Persons (N)	Person-days (N)	Daily questionnaire responses(N)	Fully vaccinated persons(N)	Negative PCR tests(N)	Positive PCR tests(N)	Daily testing rate (%)	SARS-CoV-2 infection rate per 100 000 person-days
Invited with follow-up data ^a^	25 365	3 253 671		17 815	177 511	929	5.5	28.6
Participants ^b^	6337	753 607	471 986	5082	53 266	213	7.1	28.3
Missing data on COVID-19 contact ^c^	352	239 442	23 238	261	17 270	54	7.1	22.6
Study population	5985	514 165	448 748	4821	35 996	159	7.1	30.9

a Follow-up from 25 November 2020 until the first positive PCR test, 7 days after full vaccination or 30 April 2021.

b Follow-up from 7 days after first questionnaire response until first positive PCR test, 7 days after full vaccination or 30 April 2021.

c Person-days at risk with missing information on close contact 3–7 days earlier with patients, co-workers or persons outside work with COVID-19 that were not included in the analyses.

Table 2 presents characteristics (person-days) of the invited population and the study population by COVID-19 contacts 3–7 days earlier. The study population included 88.6% women and the mean age was 48.0 years compared with 83% women and a mean age of 43.6 years for the invited population. Compared to the invited healthcare workers, more study participants had been PCR tested earlier. Only minor occupation and department differences between the invited and the participating populations were seen, except for relatively more participants from departments with less frequent patient contact.

**Table 2:** Population characteristics (person-days) according to participation status and contact 3–7 days earlier with patients, co-workers and persons outside work with COVID-19. [COPD=chronic obstructive pulmonary disease]

Characteristics	Invited population	COVID-19 contact among participants					

		Patients		Co-workers		Persons outside work	
			
	N=3 253 671Mean age 43.4 (SD 12.1) years	No (N=488 147)Mean age 49.5 (SD 10.3) years	Yes (N=26 018)Mean age 47.3 (SD 11.1) years	No (N=510 012)Mean age 49.4 (SD 10.4) years	Yes (N=4153)Mean age 49.2 (SD 10.6) years	No (N=509 358)Mean age 49.4 (SD 10.4) years	Yes (N=4 807)Mean age 48.4 (SD 10.5) years
						
	N (%)	N (%)	N (%)	N (%)	N (%)	N (%)	N (%)
Women	2 708 710 (83)	435 986 (89)	22 650 (87)	455 003 (89)	3633 (87)	454 360 (89)	4276 (89)
COVID-19 contact							
Patients				24 658 (5)	1360 (33)	25 504 (5)	514 (11)
Co-workers		2793 (1)	1360 (5)			4039 (1)	114 (2)
Persons outside work		4293 (1)	514 (2)	4693 (1)	114 (3)		
Months							
November	760 392 (23)	15 286 (3)	922 (4)	16 039 (3)	169 (4)	16 005 (3)	203 (4)
December	571 757 (18)	129 414 (27)	9527 (37)	136 712 (27)	2229 (54)	135 885 (27)	3056 (64)
January	535 177 (16)	126 987 (26)	11 459 (44)	136 899 (27)	1547 (37)	137 437 (27)	1009 (21)
February	456 640 (14)	85 888 (18)	2507 (10)	88 306 (17)	89 (2)	88 222 (17)	173 (4)
March	152 046 (5)	73 007 (15)	913 (4)	73 861 (14)	59 (1)	73 759 (14)	161 (3)
April	777 659 (24)	57 565 (12)	690 (3)	58 195 (11)	60 (1)	58 050 (11)	205 (4)
PCR tests (1–2 days earlier)							
0	2 909 178 (89)	421 861 (86)	21 117 (81)	440 408 (86)	2570 (62)	439 936 (86)	3042 (63)
1–2	343 106 (11)	66 108 (14)	4808 (19)	69 362 (14)	1554 (38)	69 176 (14)	1740 (37)
PCR tests (3–7 days earlier)							
0	2 460 903 (76)	334 339 (68)	15 461 (59)	347 771 (68)	2029 (49)	347 449 (68)	2351 (49)
1	753 731 (23)	147 119 (30)	9269 (36)	154 621 (30)	1767 (43)	154 484 (30)	1904 (40)
≥2	39 037 (1)	6 689 (1)	1288 (5)	7620 (1)	357 (9)	7425 (1)	552 (11)
PCR tests (≥ 8 days earlier)							
0	647 158 (20)	53 070 (11)	1921 (7)	54 586 (11)	405 (10)	54 415 (11)	576 (12)
1–4	1 346 111 (41)	215 792 (44)	11 404 (44)	224 818 (44)	2378 (57)	224 339 (44)	2857 (59)
5–9	861 939 (26)	141 699 (29)	9720 (37)	150 303 (29)	1116 (27)	150 374 (30)	1045 (22)
≥10	398 463 (12)	77 586 (16)	2973 (11)	80 305 (16)	254 (6)	80 230 (16)	329 (7)
Occupation							
Nursing staff ^a^	1 264 494 (39)	180 659 (37)	13 532 (52)	192 065 (38)	2126 (51)	192 077 (38)	2114 (44)
Medical doctors	451 218 (14)	43 574 (9)	3092 (12)	46 275 (9)	391 (9)	46 184 (9)	482 (10)
Biomedical Laboratory	156 073 (5)	36 657 (8)	3398 (13)	39 788 (8)	267 (6)	39 769 (8)	286 (6)
Medical secretaries	277 011 (9)	61 854 (13)	864 (3)	62 357 (12)	361 (9)	62 091 (12)	627 (13)
Other	1 094 735 (34)	165 260 (34)	5127 (20)	169 379 (33)	1008 (24)	169 089 (33)	1298 (27)
Missing	10 140 (0)	143 (0)	5 (0)	148 (0)	0 (0)	148 (0)	0 (0)
Department							
Emergency	120 912 (4)	9 413 (2)	2509 (10)	11 602 (2)	320 (8)	11 686 (2)	236 (5)
Medicine ^b^	776 923 (24)	123 542 (25)	6557 (25)	128 932 (25)	1167 (28)	128 827 (25)	1272 (26)
Surgery ^c^	603 097 (19)	86 542 (18)	2897 (11)	88 877 (17)	562 (14)	88 581 (17)	858 (18)
Biochemistry	184 158 (6)	38 959 (8)	3273 (13)	42 018 (8)	214 (5)	41 942 (8)	290 (6)
Service ^d^	109 284 (3)	6 509 (1)	651 (3)	7104 (1)	56 (1)	7 137 (1)	23 (0)
Anaesthesiology	123 557 (4)	16 121 (3)	3325 (13)	19 169 (4)	277 (7)	19 246 (4)	200 (4)
Radiology and nuclear medicine	134 114 (4)	21 574 (4)	1500 (6)	22 826 (4)	248 (6)	22 821 (4)	253 (5)
Psychiatry	427 205 (13)	61 721 (13)	1266 (5)	62 474 (12)	513 (12)	62 343 (12)	644 (13)
Departments with less frequent patient contact ^e^	455 684 (14)	92 302 (19)	2483 (10)	94 281 (18)	504 (12)	94 051 (18)	734 (15)
Other ^f^	308 597 (9)	31 321 (6)	552 (6)	32 581 (6)	292 (7)	32 576 (6)	297 (6)
Missing	10 140 (0)	143 (0)	5 (0)	148 (0)	(0)	148 (0)	0 (0)
Smoking							
Current smoker		26 498 (5)	1951 (7)	28 222 (6)	227 (5)	28 150 (6)	299 (6)
Previous smoker		142 411 (29)	7660 (29)	148 682 (29)	1389 (33)	148 549 (29)	1522 (32)
Never smoker		315 544 (65)	16 251 (62)	329 287 (65)	2508 (60)	328 822 (65)	2973 (62)
Missing		3694 (1)	156 (1)	3821 (1)	29 (1)	3837 (1)	13 (0)
BMI (kg/m^2^)							
<20		32 317 (7)	1710 (7)	33 764 (7)	263 (6)	33 715 (7)	312 (6)
20–24		228 612 (47)	11 608 (45)	238 506 (47)	1714 (41)	237 771 (47)	2449 (51)
25–29		146 236 (30)	7777 (30)	152 586 (30)	1427 (34)	152 662 (30)	1351 (28)
≥30		77 116 (16)	4734 (18)	81 143 (16)	707 (17)	81 168 (16)	682 (14)
Missing		3 866 (1)	189 (1)	4013 (1)	42 (1)	4042 (1)	13 (0)
Lung disease							
Hay fever		100 096 (21)	4827 (19)	104 107 (20)	816 (20)	103 924 (20)	999 (21)
Asthma		34 659 (7)	1668 (6)	36 036 (7)	291 (7)	35 900 (7)	427 (9)
COPD		3091 (1)	188 (1)	3232 (1)	47 (1)	3231 (1)	48 (1)

a Nurses, social- and healthcare assistants and radiographers

b Internal medicine, paediatrics, oncology and neurology

c All surgical departments, including: obstetrics and gynaecology; otorhinolaryngology, head and neck surgery; and ophthalmology

d Cleaning services; hospital porters; clothing and waste management; depot and archive; telephone switchboard; and guidance for patients, relatives and staff

e Occupational and social medicine; physio- and occupational therapy; administration; department of technical services; and kitchen

f Administrative, technical and pedagogical staff

Participants who reported one type of close COVID-19 contact more often reported the other types of close COVID-19 contact. All types of COVID-19 contact were associated with more frequent PCR testing, especially during the previous 1–2 days. More nurses had close contact with patients and co-workers with COVID-19 than other occupations. Only small differences were seen for department, smoking status, BMI and lung diseases.

After having close contact with COVID-19 patients 3–7 days earlier, 40 participants tested positive for SARS-CoV-2, while 119 tested positive after no such known contact (table 3). This corresponded with infection rates of 153.7 and 24.4 per 100 000 person-days and an adjusted IRR of 3.17 (95% CI 2.15–4.66). After having close contact with co-workers and persons outside work with COVID-19 3-7 days earlier, 10 and 35 participants tested positive corresponding with infection rates of 240.8 and 728.1 per 100 000 person-days, respectively. The infection rates among those with no such known contacts were 29.2 and 24.3 per 100 000 person-days and the adjusted IRR were 2.54 (95% CI 1.30–4.96) and 17.79 (95% CI 12.05–26.28), respectively.

**Table 3:** Close contact 3–7 days earlier with patients, co-workers and persons outside work with COVID-19 and incidence rate ratios (IRR) of SARS-CoV-2. [CI=confidence interval.]

Contact with persons with COVID-19	Person-days	First positive SARS-CoV-2 PCR tests	Infection rate per 100,000 person-days	Model 1	Model 2 ^a^	Model 3 ^b^	Model 4 ^c^
			
				IRR (95% CI)	IRR (95% CI)	IRR (95% CI)	IRR (95% CI)
Patients							
No contact	488 147	119	24.4	Reference	Reference	Reference	Reference
Contact	26 018	40	153.7	6.31 (4.41–9.02)	4.62 (3.21–6.65)	3.72 (2.55–5.44)	3.17 (2.15–4.66)
Co-workers							
No contact	510 012	149	29.2	Reference	Reference	Reference	Reference
Contact	4153	10	240.8	8.24 (4.34–15.63)	5.44 (2.86–10.35)	2.68 (1.37–5.24)	2.54 (1.30–4.96)
Persons outside work							
No contact	509 358	124	24.3	Reference	Reference	Reference	Reference
Contact	4807	35	728.1	29.91 (20.55–43.52)	21.75 (14.75–32.06)	18.87 (12.78–27.88)	17.79 (12.05–26.28)

a Model 1 adjusted for age (continuous), sex and month (6 categories, November 2020-April 2021)

b As model 2 and additionally adjusted for the other types of COVID-19 contact

c As model 3 and additionally adjusted for number of previous PCR tests.

When comparing the risk of SARS-CoV-2 infection following close contact with either patients or co-workers with close contact with persons outside work with COVID-19, excluding person-days and cases with combined exposures, we observed IRR of 0.17 (34 cases, 95% CI 0.10–0.28) for patients and 0.21 (5 cases, 95% CI 0.08–0.54) for co-workers (table 4). This analysis included 34 cases in the reference group with COVID-19 contact outside work.

**Table 4:** Incidence rate ratios (IRR) of SARS-CoV-2 following close contact 3-7 days earlier with either patients or co-workers compared with contact with persons outside work with COVID-19.^a^[CI=confidence interval.]

Contact with persons with COVID-19	Person-days	First positive SARS-CoV-2 PCR tests	Infection rate per 100 000 person-days	Model 1	Model 2 ^b^	Model 3 ^c^
		
				IRR (95% CI)	IRR (95% CI)	IRR (95% CI)
Patients	24 203	34	140.5	0.18 (0.11–0.28)	0.16 (0.10–0.27)	0.17 (0.10–0.28)
Co-workers	2 738	5	182.6	0.23 (0.09–0.58)	0.21 (0.08–0.54)	0.21 (0.08–0.54)
Persons outside work	4 238	34	802.3	Reference	Reference	Reference

a Only person-days at risk with contact either with patients, co-workers or persons outside work with COVID-19 are included

b Model 1 adjusted for age (continuous), sex and month (6 categories, November 2020-April 2021)

c As model 2 and additionally adjusted for number of previous PCR tests.

A total of 24 participants with incident loss of taste and smell had experienced close contact with COVID-19 patients (table 5). This corresponded with an IRR of 41.4 per 100 000 person-days and an adjusted IRR of 1.48 (95% CI 0.95–2.29). Following close contact with co-workers and persons outside work with COVID-19, the adjusted IRR of loss of taste and smell were 2.56 (95% CI 1.24–5.30) and 10.82 (95% CI 7.33–15.98). Among those reporting loss of taste and smell, 36% had a positive PCR test earlier.

**Table 5:** Close contact 3–7 days earlier with patients, co-workers and persons outside work with COVID-19 and incidence rate ratios (IRR) of loss of taste and smell. [CI=confidence interval.]

Close contact with persons with COVID-19	Person-days ^a^	Incident loss of taste and smell	Incidence rate per 100,000 person-days	Model 1	Model 2 ^b^	Model 3 ^c^
		
				IRR (95% CI)	IRR (95% CI)	IRR (95% CI)
Patients						
No contact	488 451	202	41.4	Reference	Reference	Reference
Contact	26 748	24	89.7	2.17 (1.42–3.31)	1.70 (1.11–2.61)	1.48 (0.95–2.29)
Co-workers						
No contact	511 010	218	42.7	Reference	Reference	Reference
Contact	4189	8	191.0	4.48 (2.21–9.07)	3.20 (1.57–6.49)	2.56 (1.24–5.30)
Persons outside work						
No contact	510 123	195	38.2	Reference	Reference	Reference
Contact	5076	31	610.7	15.98 (10.94–23.34)	11.21 (7.60–16.54)	10.82 (7.33–15.98)

a This population was slightly different from that of table 3 because of the different outcome.

b Adjusted for age (continuous), sex and month (6 categories, November 2020-April 2021).

c As model 2 and additionally adjusted for the other types of COVID-19 contact.

The infection rate in the study population declined from January to April 2021, increased by number of PCR tests 3–7 days earlier and were higher for departments of medicine and among nurses compared with other departments and occupations. No clear infection rate patterns were seen for the other population characteristics (supplementary table S1).

Participants reported an overall 2% non-compliance with PPE guidelines during 187 413 daily procedures. For respiratory procedures with potential for higher exposure levels, this percentage was 4.8% (supplementary table S2).

Sensitivity analyses that only included COVID-19 contact information obtained before results of the PCR tests were available, showed an infection rate of 155.2 per 100 000 person-days and an adjusted IRR of 3.52 (95% 2.41–5.13) following close contact with COVID-19 patients (supplementary table S3). The IRR following close contact with persons outside work with COVID-19 was 14.19 (95% CI 8.27–24.33). No results were available for close contact with co-workers with COVID-19 because this information was obtained after PCR test results were available for those tested.

## Discussion

### Principal findings

This follow-up study of healthcare workers was conducted from 25 November 2020 to 30 April 2021 during the second wave of the pandemic in Denmark. The SARS-CoV-2 infection rates following close contact with patients, co-workers, and persons outside work with COVID-19 were, respectively, 153.7, 240.8 and 728.1 per 100 000 person-days. This corresponded to about 3, 2.5 and 18-fold increased adjusted IRR when compared with no such contacts representing the background risk. When compared with close contacts outside work, the adjusted IRR of close contacts with patients and co-workers with COVID-19 were about 5-fold decreased. Among all healthcare workers, the absolute numbers affected following close contact with patients or co-workers was higher than the absolute numbers following close contact with persons outside work with COVID-19. Comparable patterns of increased risks of loss of taste and smell were seen for all three types of COVID-19 contact. Participants reported high but not complete day-to-day compliance with PPE guidelines.

### Strengths and limitations

Strengths of this study are the follow-up design with day-to-day information that allowed precise account for incubation period and daily change in exposure, the complete follow-up for PCR test results, and information on incident loss of taste and smell that was a key symptom of SARS-CoV-2 infection (22). During spring 2020, in this population, persistent loss of taste and smell was strongly associated with a positive PCR test for SARS-CoV-2 with an odds ratio of 57.16 (95% CI 16.71–195), corresponding with a specificity of 98% and a positive predictive value of 84% (16). Other strengths are the free access to PCR testing and the high testing rate. The decision to be PCR tested was therefore unlikely to be strongly associated with COVID-19 contact and result of the PCR test, and we regard collider bias a minor problem (23).

Participants reporting one type of close COVID-19 contact (patients, co-workers or persons outside work) more often experienced the other types of contact and the mutually adjusted IRR estimates were substantially reduced and are expected to provide the best estimates of the separate effects, supported by our finding in the direct comparison between the three types of contact.

Participants with close COVID contacts had been PCR tested for SARS-CoV-2 infection more often than those with no such contacts. However, earlier PCR tests (all negative) should not be causally associated with SARS-CoV-2 infection as detected by a positive PCR test on a given day of follow-up but may be indicators of unobserved risk factors of SARS-CoV-2 infection that may confound associations. Our analyses indicated no such confounding. Furthermore, analyses of loss of taste and smell, which is a key symptom of SARS-CoV-2 infection unaffected by PCR testing frequency, showed results in line with those seen for SARS-CoV-2 infection.

Participants were about five years older than the invited population. The risk of SARS-CoV-2 infection has been suggested to increase with age, but we previously observed the highest SARS-CoV-2 sero-prevalence among the young participants of 18 000 healthcare workers from the source population of this study (24, 25). Participation was higher among healthcare workers from departments with less frequent patient contact, and we thus have no obvious explanation for the slightly higher incidence rate of SARS-CoV-2 infection in the study compared to source population.

Analyses were mutually adjusted for other COVID-19 contact forms, sex, age, month and number of PCR tests. The likelihood of exposure to patients and co-workers with COVID-19 varied across departments and occupations, but because we had individual information on contact with patients and co-workers with COVID-19 for each day of follow-up, department and occupation were not included in the adjusted models.

COVID-19 contact information was partly obtained after the results of the PCR test results were available for the tested participants, which may have introduced recall bias and inflated results. However, sensitivity analyses relying only on contact information obtained before results of the PCR tests were available indicated no substantial recall bias. Knowledge of PCR test results as well as COVID-19 contact may, on the other hand, have inflated results for loss of taste and smell.

Being classified with no close COVID-19 contact during the 5-day exposure window allowed missing information for two of the five days. Because there may have been COVID-19 contact during these days, this may have attenuated IRR.

Our study population included mainly hospital healthcare workers, and findings may be less representative for healthcare workers in primary care because overall incidence of SARS-CoV-2 infection has been reported lower for general practitioners (3.50%) and nursing home staff (3.92%) than for hospital workers (5.32%) when the second wave of the pandemic peaked in Denmark in December 2020 (26).

### Comparisons with other studies

Numerous studies during the last two years have documented increased risk and mortality of COVID-19 as well as hospitalization among healthcare workers as shown by several reviews (4, 6, 16). Lower risk for healthcare workers during the second than the first wave of the pandemic has been shown (27).

This study showed an overall SARS-CoV-2 infection rate of 30.9 per 100 000 person-days during the second wave, which was well below the self-reported positive PCR testing rate of 132 per 100 000 person-days observed in a prospective cohort of frontline healthcare worker during the first wave by Nugyen et al (2). Our observed infection rates of 153.7 and 240.8 per 100 000 person-days following contact with patients and colleagues with COVID-19 were, however, slightly higher than the infection rates of about 90 and 70 per 100 000 person-days reported by Mo et al (7) for healthcare workers with similar contacts at UK hospitals during the first wave. On the other hand, Nguyen et al (2) reported a higher infection rate of 553 per 100 000 person-days following contact with COVID-19 patients for healthcare workers reporting that they always had the PPE they needed (with no further specification).

We observed a SARS-CoV-2 infection rate of 728.1 per 100 000 person-days following close contact with persons outside work with COVID-19, which was half the average household infection rate of 1660 per 100 000 person-days reported for the first wave (12). This may partly reflect that we included any close contact with a person outside work with COVID-19 and not only household contacts that are expected to be closer and last longer.

Several serological studies have provided results in line with ours for contact with patients (8–11), co-workers (8, 9) and persons outside work with COVID-19 (8, 9, 12–15). But there are also findings of no association (13–16).

The findings of these studies are, however, not directly comparable because of differences in population compositions, background incidence rates, definitions of COVID-19 contact and SARS-CoV-2 infection. We are not aware of other studies comparing the incidence rates of SARS-CoV-2 infection following exposure to patients or co-workers at work with persons outside work with COVID-19.

During a 17-day period around New Year 2021, a subset of the healthcare workers of the current study had used a new respirator with frequent defects during contact with COVID-19 patients that may partly have contributed to the increased risk we observed (28).

### Concluding remarks

During the second wave of the pandemic, this healthcare worker population was at increased risk of SARS-CoV-2 infection when in close contact with patients and colleagues with COVID-19, but the risk was not as high as after contact with persons outside work with COVID-19. However, the absolute numbers affected following contact with patients and co-workers were higher than the absolute numbers affected following COVID-19 contact outside work. PPE was not in shortage in the healthcare sector, guidelines for PPE use and other infection control measures were implemented but did not include a fit test. Compliance with required PPE was high but not complete.

The current findings thus stress the need for increased focus on use of recommended PPE, correct donning, doffing, fit test and other procedures (18, 29–31), training (32) and ventilation (33). The aim is to secure healthcare workers’ health and reduce transmission into the community (34) during ongoing and future waves of SARS-CoV-2 and other infections.

## Supplementary material

Supplementary material
